# Mechanical Metrics of the Proximal Tibia are Precise and Differentiate Osteoarthritic and Normal Knees: A Finite Element Study

**DOI:** 10.1038/s41598-018-29880-y

**Published:** 2018-07-31

**Authors:** Hanieh Arjmand, Majid Nazemi, Saija A. Kontulainen, Christine E. McLennan, David J. Hunter, David R. Wilson, James D. Johnston

**Affiliations:** 1 Department of Mechanical Engineering, University of Saskatchewan, Saskatoon, SK Canada; 2 College of Kinesiology, University of Saskatchewan, Saskatoon, SK Canada; 3 Division of Research, New England Baptist Hospital, Boston, MA USA; 4 Institute of Bone and Joint Research, Kolling Institute, University of Sydney and Rheumatology Department, Royal North Shore Hospital, Sydney, NSW Australia; 5 Department of Orthopaedics and Centre for Hip Health and Mobility, University of British Columbia and Vancouver Coastal Health Research Institute, Vancouver, BC Canada

**Keywords:** Biomedical engineering, Computational science

## Abstract

Our objective was to identify precise mechanical metrics of the proximal tibia which differentiated OA and normal knees. We developed subject-specific FE models for 14 participants (7 OA, 7 normal) who were imaged three times each for assessing precision (repeatability). We assessed various mechanical metrics (minimum principal and von Mises stress and strain as well as structural stiffness) across the proximal tibia for each subject. *In vivo* precision of these mechanical metrics was assessed using CV%_RMS_. We performed parametric and non-parametric statistical analyses and determined Cohen’s *d* effect sizes to explore differences between OA and normal knees. For all FE-based mechanical metrics, average CV%_RMS_ was less than 6%. Minimum principal stress was, on average, 75% higher in OA versus normal knees while minimum principal strain values did not differ. No difference was observed in structural stiffness. FE modeling could precisely quantify and differentiate mechanical metrics variations in normal and OA knees, *in vivo*. This study suggests that bone stress patterns may be important for understanding OA pathogenesis at the knee.

## Introduction

Altered subchondral bone (bone below cartilage) in the early stages of osteoarthritis (OA) is thought to play a major role in cartilage degradation, OA development, and OA-related pain^1–4^. OA alters morphology and biomechanical properties of bone. Morphological alterations include subchondral bone sclerosis, cyst formation within the subchondral trabecular bone, and osteophyte formation along the joint periphery^5^. Biomechanical alterations include altered mechanical stiffness of the subchondral bone surface^6–9^ and altered bone tissue stiffness^10–12^. Excessive bone mechanical stress caused by OA-related bone alterations may initiate microdamage in bone leading to an increased bone turnover and accelerated OA^13^. Altered stress and strain distributions in osteoarthritic bone can result in abnormal bone remodeling as well^14^. Additionally, given that cartilage is aneural (lacking nerves) whereas bone is highly innervated^15,16^, bone is a potential initiatory site of pain with altered bone morphology and mechanics as the source^17–20^. Although the importance of subchondral bone in OA development and progression is commonly accepted in the literature, current theories for the structural role of subchondral bone in OA are mostly based on animal or *ex vivo* cadaveric studies. Since various OA factors (e.g., physical activity, alignment) and features (e.g., OA-related pain) are typically unknown in animal and *ex vivo* cadaveric studies, accurate *in vivo* analyses are needed.

Finite element (FE) modeling is a non-invasive method that has been used to investigate associations between OA-related morphological and mechanical property alterations in bone and overall joint mechanics. Although FE modeling has the potential to assess bone mechanical behavior (i.e., stress, strain, stiffness) *in vivo*, most OA-related FE research to date was conducted with simplified geometry, loading, material properties, or with simulated defects in the bone^4,21–24^. These studies test whether altered subchondral morphology and mechanical properties, cyst formation, and alignment changed stress/strain distributions and structural stiffness of bone. Although the previous research has suggested a potential structural role of bone in OA initiation and development, most FE research-to-date has been simulation-based with a focus on individual alterations (e.g., studying the effect of cyst size on the stress levels by McErlain *et al*.^22^ and studying the effects of subchondral bone and cartilage stiffness on the stress levels by Dar and Aspden^24^). To examine the combined effects of these individual bone alterations (i.e., bone mineral density (BMD) distribution, alignment, presence of cysts, etc.) and represent accurate bone geometry and material properties, we need to develop subject-specific FE models that can incorporate various bone alterations. Quantitative computed tomography images (QCT) are commonly used to create subject-specific FE models^22,25–27^. In this method, the geometry of the bony tissue is obtained using segmented QCT images and image-based densities are converted to elastic moduli using published density-modulus relationships. With subject-specific FE modeling, we can assess various mechanical metrics of bone (which cannot be measured otherwise), and study them in OA and normal knees. However, information regarding precision (i.e., repeatability) errors for FE-based mechanical metrics is important when applying these methods to analyze and compare OA and normal knees.

**Figure 1:**
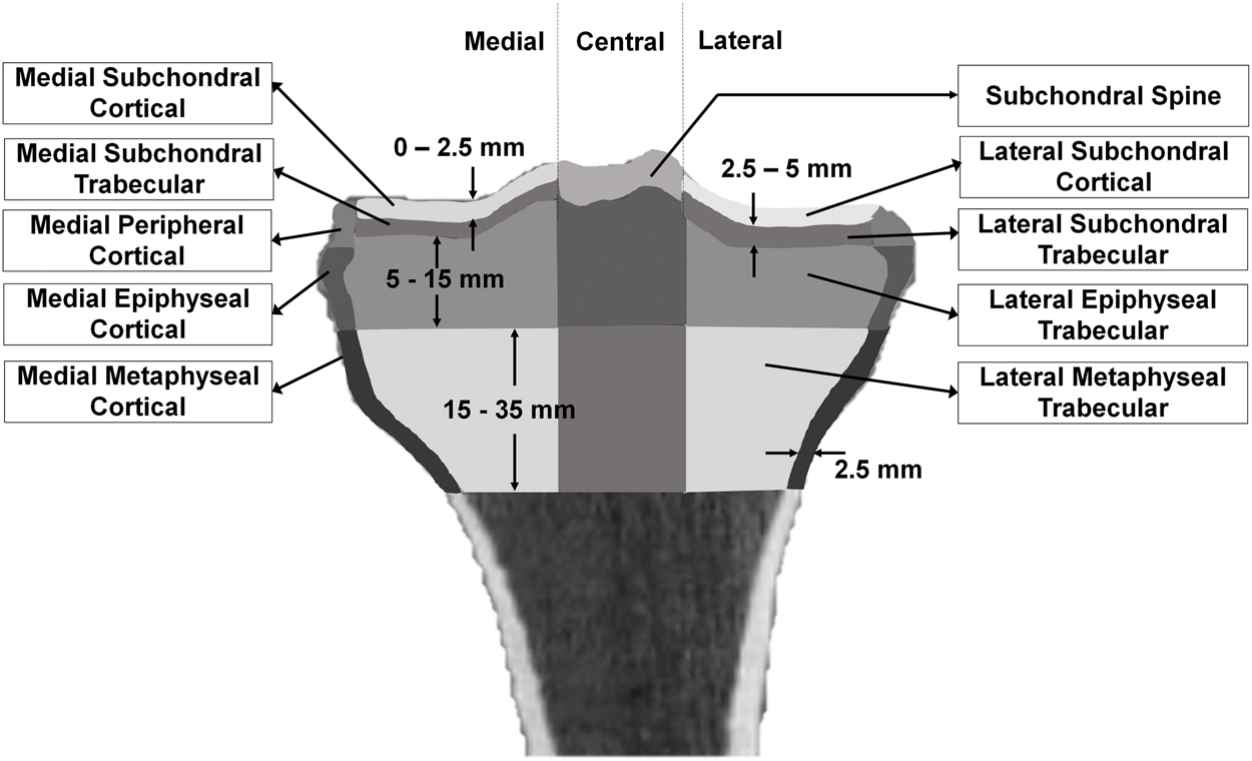
Regions used for analyzing the proximal tibia metrics. Images shows the different regions used for analyzing FE result of the proximal tibia. Lateral regions are located on the right side of the image while medial regions are at the left side of the image.

Our objective was to identify precise mechanical metrics of the proximal tibia that differentiated OA and normal knees. For this, we used QCT scans of the knees of fourteen participants. Seven of the knees were classified as normal while the remaining seven were classified as having OA (details of participant and image acquisition is provided in the Methods section). Each knee was imaged three times for the purpose of calculating precision errors. Subject-specific FE models of the knees were created, and single-leg stance loading was simulated (see details in Method section). FE-based minimum principal and von Mises stress and strain were obtained, as well as medial and lateral proximal tibial stiffness. Regional stress and strain were assessed across the proximal tibia (Fig. 1). Short-term precision errors were assessed by calculating root mean square coefficients of variation (CV%_RMS_) for all outcomes and in different regions of the proximal tibia. As well, pairwise comparisons of FE-based outcomes as well as BMD, bone mineral content (BMC) and bone volume were performed between OA and normal knees.

## Results

### Precision Study

CV%_RMS_ for both OA and normal knees ranged from 3.7% to 10.5% (average: 6.1%) for minimum principal stress (Table 1) and 3.2% to 7.6% (average: 5.5%) for minimum principal strain (Table 2). Similar precision errors were noted for von-Mises stress (Supplement Table 1) and strain (Supplement Table 2). Precision error for structural stiffness for both OA and normal participants was 3.6% in the medial compartment and 5.0% in the lateral compartment (Table 3).

**Table 1:** Minimum principal stress comparison between OA and normal proximal tibia.

Minimum principal stress (MPa)	All scans		CV%_RMS_	OA knees		Normal knees		Difference		95% CI		p-value	Cohen’s *d*
	Mean	SD		Mean/Median*	SD	Mean /Median*	SD	Absolute	Percent	Lower	Upper		
Medial peripheral cortical*	**−0.36**	**0.19**	**5.7**	**−0.51**	**0.18**	**−0.22**	**0.08**	**−0.25**	**−115.3%**	**−0.36**	**−0.11**	**0.006**	**1.36**
**Medial epiphyseal cortical**	**−0.59**	**0.31**	**6.6**	**−0.80**	**0.28**	**−0.38**	**0.17**	**−0.41**	**−107.2%**	**−0.68**	**−0.14**	**0.006**	**1.33**
Medial metaphyseal cortical*	**−2.06**	**0.88**	**7.7**	**−2.56**	**0.95**	**−1.38**	**0.27**	**−1.18**	**−85.2%**	**−1.71**	**−0.22**	**0.013**	**1.28**
Medial subchondral cortical*	**−0.52**	**0.16**	**3.9**	**−0.63**	**0.18**	**−0.41**	**0.06**	**−0.17**	**−41.7%**	**−0.35**	**−0.02**	**0.018**	**1.22**
Medial subchondral trabecular*	**−0.46**	**0.15**	**3.7**	**−0.51**	**0.17**	**−0.37**	**0.06**	**−0.15**	**−41.1%**	**−0.33**	**−0.01**	**0.035**	**1.13**
Medial epiphyseal trabecular*	**−**0.43	0.15	4.8	**−**0.47	0.18	**−**0.35	0.06	**−**0.12	**−**33.2%	**−**0.32	0.01	0.085	1.03
Medial metaphyseal trabecular*	**−**0.51	0.26	8.9	**−**0.55	0.30	**−**0.34	0.10	**−**0.21	63.0%	**−**0.41	0.01	0.064	1.01
**Subchondral spine**	**−0.30**	**0.10**	**5.3**	**−0.37**	**0.07**	**−0.24**	**0.08**	**−0.14**	**−56.9%**	**−0.22**	**−0.05**	**0.005**	**1.35**
Epiphyseal central	**−**0.14	0.06	8.2	**−**0.16	0.03	**−**0.14	0.07	**−**0.02	**−**12.3%	**−**0.08	0.05	0.559	0.33
Metaphyseal central	**−**0.12	0.04	7.3	**−**0.13	0.03	**−**0.12	0.05	**−**0.01	**−**3.8%	**−**0.05	0.04	0.838	0.12
Lateral subchondral cortical	**−**0.32	0.09	5.0	**−**0.35	0.11	**−**0.29	0.06	**−**0.07	**−**22.8%	**−**0.17	0.03	0.175	0.74
Lateral subchondral trabecular	**−**0.25	0.07	4.6	**−**0.26	0.08	**−**0.25	0.06	**−**0.02	**−**6.3%	**−**0.10	0.07	0.698	0.22
Lateral epiphyseal trabecular	**−**0.19	0.05	5.3	**−**0.20	0.06	**−**0.19	0.05	**−**0.01	**−**3.0%	**−**0.07	0.06	0.847	0.11
Lateral metaphyseal trabecular	**−**0.15	0.04	10.5	**−**0.15	0.04	**−**0.16	0.06	0.00	**−**2.2%	**−**0.05	0.06	0.896	0.07
Lateral peripheral cortical	**−**0.22	0.05	5.2	**−**0.24	0.05	**−**0.20	0.04	**−**0.04	**−**22.4%	**−**0.10	0.01	0.104	0.87
Lateral epiphyseal cortical	**−**0.25	0.06	5.9	**−**0.29	0.06	**−**0.23	0.04	**−**0.06	**−**25.2%	**−**0.12	0.00	0.057	1.00
Lateral metaphyseal cortical	**−**0.70	0.21	5.2	**−**0.76	0.25	**−**0.65	0.17	**−**0.11	**−**17.1%	**−**0.36	0.14	0.346	0.53

Mean and SD of repeated scans for both OA and normal, CV%_RMS_, mean and SD for OA knees, mean and SD for normal knees, the difference between OA and normal knees (absolute and percent relative to normal), 95% confidence of interval, p-value, and effect size (Cohen’s d) of minimum principal stress in different regions of proximal tibia. Measures with significant differences are shown with bold text in the table (p-value < 0.05).

*Shows regions which were not normally distributed whereby median value used in Mann-Whitney U-tests for statistical comparison, and confidence intervals were calculated using Hodges-Lehmann estimator.

**Table 2:** Minimum principal strain comparison between OA and normal proximal tibia.

Minimum principal strain (microstrain)	All scans		CV%_RMS_	OA knees		Normal knees		Difference		95% CI		p-value	Cohen’s *d*
	Mean	SD		Mean/Median*	SD	Mean/Median*	SD	Absolute	Percent	Lower	Upper		
Medial peripheral cortical	1185	512	6.1	1135	561	1236	497	**−**101	**−**8.2%	**−**719	516	0.727	0.20
Medial epiphyseal cortical	1410	601	4.2	1435	666	1385	582	50	3.6%	**−**678	778	0.883	0.08
Medial metaphyseal cortical	1130	418	4.7	1247	433	1014	399	233	23.0%	**−**252	718	0.316	0.56
Medial subchondral cortical*	563	203	5.6	536	258	512	143	16	3.1%	**−**164	258	0.949	0.28
Medial subchondral trabecular*	770	322	6.5	743	405	627	189	118	18.8%	**−**155	636	0.338	0.64
Medial epiphyseal trabecular*	2405	900	3.7	2294	1133	1990	599	281	14.1%	**−**557	1403	0.406	0.49
Medial metaphyseal trabecular	2166	933	4.7	2361	1096	1970	771	391	19.8%	**−**712	1494	0.455	0.42
Subchondral spine*	830	403	7.2	749	544	725	186	27	3.7%	**−**282	817	0.848	0.48
Epiphyseal central	2688	879	3.2	2873	1015	2503	752	370	14.8%	**−**670	1410	0.454	0.42
Metaphyseal central	1897	789	4.9	2053	921	1741	668	312	17.9%	**−**625	1248	0.482	0.39
Lateral subchondral cortical	862	330	6.4	914	410	809	249	106	13.1%	**−**289	500	0.571	0.32
Lateral subchondral trabecular	1321	447	5.2	1495	548	1148	249	347	30.2%	**−**149	843	0.154	0.78
Lateral epiphyseal trabecular*	2508	860	3.9	2434	1006	1979	611	559	28.2%	**−**346	1735	0.180	0.71
Lateral metaphyseal trabecular	1940	829	7.5	2080	948	1800	738	280	15.6%	**−**709	1269	0.549	0.34
Lateral peripheral cortical*	1157	492	5.7	933	657	964	236	10	1.0%	**−**194	953	0.848	0.50
Lateral epiphyseal cortical	1105	403	5.9	1181	475	1030	337	151	14.7%	**−**329	631	0.506	0.37
Lateral metaphyseal cortical	670	288	7.4	656	274	685	323	**−**30	**−**4.3%	**−**378	319	0.857	0.10

Mean and SD of repeated scans for both OA and normal, CV%_RMS_, mean and SD for OA knees, mean and SD for normal knees, the difference between OA and normal knees (absolute and percent relative to normal), 95% confidence of interval, p-value, and effect size (Cohen’s d) of minimum principal strain in different regions of proximal tibia.

*Shows regions which were not normally distributed whereby median value used in Mann-Whitney U-tests for statistical comparison, and confidence intervals were calculated using Hodges-Lehmann estimator.

**Table 3:** Medial and lateral stiffness comparison between OA and normal proximal tibia.

Regional Stiffness (N/mm)	All scans		CV%_RMS_	OA knees		Normal knees		Difference		95% CI		p-value	Cohen’s *d*
	Mean	SD		Mean	SD	Mean	SD	Absolute	Percent	Lower	Upper		
Medial compartment	7708	2986	3.6	8515	3767	6902	1902	1613	23.4%	**−**1862	5088	0.332	0.54
Lateral compartment	5959	1545	5.0	6200	1201	5718	1896	482	8.4%	**−**1366	2331	0.580	0.31

Mean and SD of repeated scans for both OA and normal, CV%_RMS,_ mean and SD for OA knees, mean and SD for normal knees, the difference between OA and normal knees (absolute and percent relative to normal), 95% confidence of interval, p-value, and effect size (Cohen’s d) of structural stiffness in medial and lateral compartments.

### Preliminary Comparisons of OA and Normal FE Outcomes

**Figure 2:**
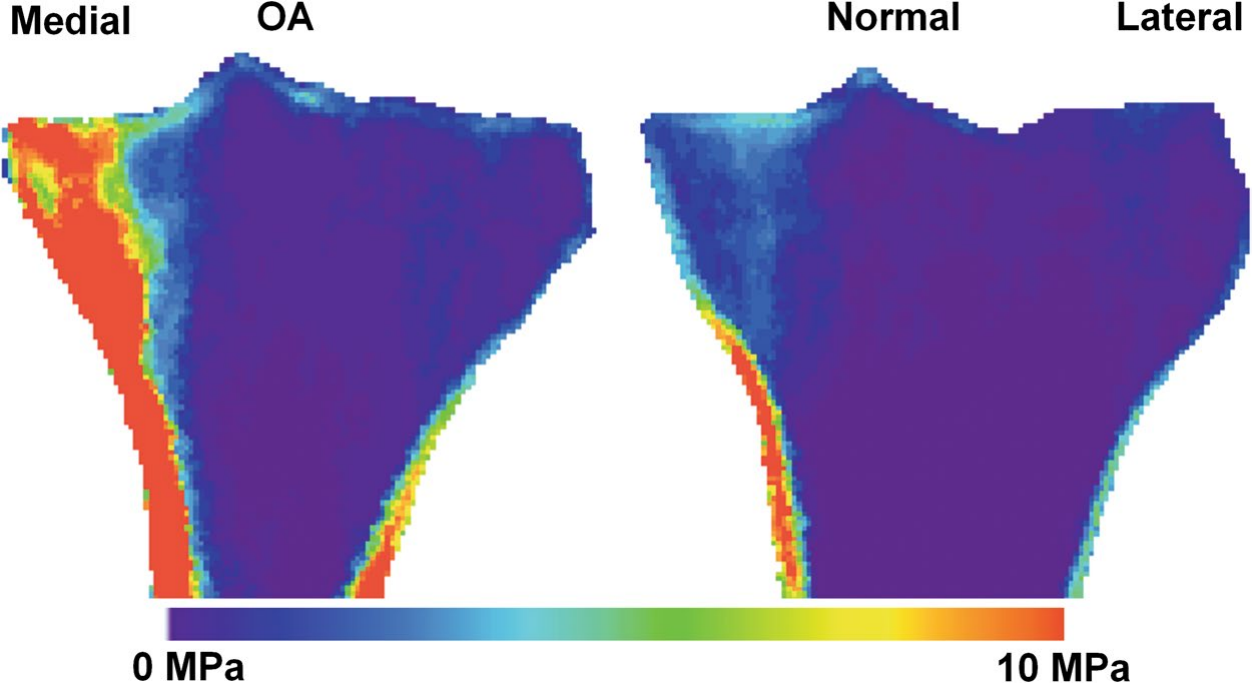
Min principal stress comparison between OA and normal proximal tibia. Min principal stress of OA and normal proximal tibia are demonstrated in coronal section of CT image. Red indicates high stress while blue is low stress.

Overall, stress (after scaling for bodyweight) was higher in OA versus normal bone. The percent difference in minimum principal stress was 115% in the medial peripheral cortical bone (~20x CV%_RMS_) and 107% in the medial epiphyseal cortical region (~16x CV%_RMS_) (Table 1). Similarly, von-Mises stress values were higher in OA knees compared to the normal knees (Supplement Table 1). Mapped representations of minimum principal stress results onto CT images of the proximal tibia are shown in Fig. 2. Qualitative analyses of the stress patterns indicated that the load was primarily transferred through the medial compartment for individuals with OA (Fig. 2).

Regarding the strain values, no difference was observed in minimum principal or von-Mises strain values between OA and normal knees (p > 0.05) (Table 2 and Supplement Table 2). The highest strain was observed in the epiphyseal central region for both normal and OA proximal tibia. Similar to the strain results, no differences were observed in the structural stiffness of medial and lateral condyles of proximal tibia between OA and normal knees (p > 0.05) (Table 3). When we compared BMD, BMC and bone volume values between OA and normal knees (Supplement Tables 3–5), no differences were noted, except BMD for the medial peripheral cortical region (p = 0.039) (Supplement Table 3).

## Discussion

This is the first study to report *in vivo* precision errors for subject-specific FE-based mechanical metrics for various regions of the proximal tibia. This is also one of few studies to differentiate OA and normal knees using FE-derived estimates of mechanical behavior. The developed FE method provided precise estimates of von-Mises stress, minimum principal stress, von-Mises strain, and minimum principal strain in the different regions of the proximal tibia as well as structural stiffness of the medial and lateral compartments (average CV%_RMS_ < 6%). Our precision errors were in the same range as previously reported CV%_RMS_ precision errors for FE modeling using high-resolution peripheral QCT imaging (HR-pQCT) to measure bone stiffness and stress^28–30^.

On average, minimum principal stress was 75% larger in the medial side of the OA bone compared to normal bone. The difference in stress was as high as 20 times the precision error for minimum principal stress, indicating that the difference is far greater than repeatability error. These findings, combined with large effect sizes (Cohen’s *d*) suggest that FE-based mechanical metrics can differentiate OA and normal knees. On a related note, we also calculated the *in vivo* precision of regional BMD, BMC and bone volume measures of the proximal tibia and when we compared these values between OA and normal knees, no differences were noted (except for BMD for the medial peripheral cortical region). This further emphasizes the importance of exploring the combined effects of individual OA-related bone alterations.

The obtained mechanical metrics are in agreement with previously published FE analyses^22,26,31^. The reported von-Mises stress values were within the same range (around 0.9 MPa with one body weight for the medial subchondral cortical region) as previous subject-specific FE modeling of the knee joint by McErlain *et al*.^22^ and an FE study of the proximal tibia by Tuncer *et al*.^26^ with similar loading conditions. Also, the obtained strain values in this research (around 700 microstrain with one body weight for the medial subchondral trabecular region) compared favorably with the strain range reported for FE modeling of the proximal tibia^26^. Structural stiffness values for the proximal tibia were similar (~10% difference) to the stiffness reported in a previous study by Amini *et al*. (8703 N/mm)^31^.

Although speculative, it is worthwhile hypothesizing on why stress was higher in OA knees as this may provide a better understanding of the mechanical behavior of bone in response to OA. We believe stress was higher due to the combined effects of various factors, including: (1) higher weight; (2) malalignment; and (3) BMD difference between OA and normal knees.

First, OA participants in this study had significantly higher weight compared to the healthy participants (+40%, p-value < 0.05), which could contribute to the observed higher stress in OA knees. Though, it is important to note that the difference in weight of OA and normal groups is much lower than the observed differences in stress values (up to +115%). Second, with regards to alignment, OA knees were slightly varus which appeared to contribute to higher stress values in the medial side of the OA proximal tibia. Third, although the differences in BMD values were not significant, there was a trend for higher BMD with OA proximal tibiae (Supplement Table 3). Because Goulet’s equation for material mapping relates BMD to elastic modulus by a power of 2.1^32^, a slightly higher BMD may lead to significant differences in elastic modulus. Importantly, higher elastic moduli will change how load is distributed through the tibia, with highest elastic moduli carrying the most load. Accordingly, higher elastic moduli will lead to higher local stress. Related to this point, simply adjusting all density measures by a power of 2.1 did not differentiate OA and normal knees (Supplement Table 6). It is important to be cognizant that, with subject-specific FE modeling, individual factors such as higher weight, altered alignment and altered BMD are inherently integrated, thereby providing insight regarding their combined effect. Statistical approaches could be used to account for these factors to derive surrogate estimates of stress; however, they could be erroneous due to the employed assumptions (e.g., independency of observations, linear vs. non-linear associations, distribution of residuals). Also, statistical approaches typically require large datasets whereas subject-specific FE modeling can be employed at the individual-level.

Interestingly, while stress was different between two groups, no difference was observed in the strain values. Similar strain levels could be an indication of bone adaptation in response to altered loading in the OA joint^33^. Further research with individuals at early stages of OA is needed to assess whether this is a progression-specific phenomenon.

This study had certain strengths worthy of discussion. First, this study met the conservative number of the patients and repeated scans per patient, as proposed by Gluer *et al*., which allowed us to establish reliable precision errors with an upper 90% confidence limit having less than 30% error (e.g., if the precision error was 2%, then we were 90% confident that the true precision error is less than 2.6%)^34^. Second, we assessed *in vivo* precision errors in order to incorporate uncertainty associated with subject motion. This approach offers a more realistic estimate of repeatability error than an *ex vivo* precision study of motionless cadavers or repeated analyses of a single scan. Third, we used CT images of study participants to create FE models as opposed to idealized geometries that resemble the knee joint. Fourth, the heterogeneity of bone mechanical properties was considered using a density-modulus equation which has been shown to affect the accuracy of QCT-based FE models^25,35^. Fifth, we aligned all knees in similar 3D orientations relative to landmark boundary points and best-fit planes. This minimized differences due to dissimilar orientations during image acquisition, thereby permitting dependable comparisons between OA and normal FE outcomes. Sixth, we provided precision errors for each mechanical metric as well as differences between OA and normal knees. As these differences were, on average, 14 times greater than precision errors, these differences are trustworthy.

Limitations of this study pertain to small sample size for statistical comparison, the use of a single density-modulus equation for both cortical and trabecular bone, and simplified modeling of cartilage and menisci. First, although the number of study participants was sufficient for calculating precision errors, we had low statistical power for detecting differences between OA and normal knees. As this was a preliminary study to detect potential differences between knees, we believe the use of a small sample size for statistical comparisons was justified. Further analyses with a larger sample size are needed to corroborate study findings. Second, a single density-modulus equation was used to derive elastic moduli for both cortical and trabecular bone in the FE models. While applying cortical-specific and trabecular-specific density-modulus relationships offers improved predictions of stiffness (R^2^ = 0.77 versus R^2^ = 0.70 with a single equation), these improvements are moderate. As such, we believe a single equation is justified to provide reasonable estimates of proximal tibial mechanical behavior^25,36^. Related to this point, we did run a pilot study with cortical-specific and trabecular-specific equations for 3 samples and FE outcomes were similar to those reported with a single equation. For this reason, we applied the simpler single-equation approach for converting BMD to E. Third, as only CT images were available, realistic subject-specific modeling of cartilage and menisci was not feasible. To overcome this limitation, a cylinder of tissue with an isotropic homogeneous material was used to model soft tissue of the knee joint, as per McErlain *et al*.^22^, with the same material properties for both OA and normal knees.

Our approach to model soft tissues has both negative and positive aspects. As participants were not bearing weight during scanning, our model of soft tissue may not mimic the relative thickness of medial versus lateral cartilage, which could lead to erroneous estimates of how load and stress is distributed through the proximal tibia. On the other hand, without weight bearing our model likely overestimates cartilage thickness. This is important because prior research indicates that thinner cartilage leads to higher bone stress^24^. The approach employed for modeling cartilage also ignores cartilage defects seen with OA^37^, which would lead to a smaller contact area and higher bone stress in the subchondral cortical region. Accordingly, we believe this approach makes our analysis conservative. In other words, if subject-specific cartilage geometry and material properties were used, we would reasonably expect to see higher differences in stress between OA and normal bone due to thinner cartilage and the smaller contact area between articulating bones. Also, incorporation of estimated cartilage and meniscal geometry would have added new variability to the model. As this analysis was focused on bone, we aimed to create a reasonable, functional *in vivo* model of the knee which could be used to investigate the structural role of bone in OA. We believe we have achieved that aim; though, further research and development are needed to incorporate cartilage and meniscal structures with subject-specific geometry and mechanical properties (most likely via registration with magnetic resonance (MR) images) and lessen model development time (each model takes approximately 3 hours to develop and process).

Results of this study indicate that subject-specific FE modeling has potential to precisely quantify and differentiate mechanical metrics variations in normal and OA knees, *in vivo*. Preliminary findings suggest that OA bone exhibits higher stress levels compared to normal bone. In contrast, strain and structural stiffness did not differentiate between OA and normal bone. Subject-specific FE modeling may reveal insight into the structural role of bone in OA pathogenesis and warrant application in future studies.

## Methods

### Study Participants and OA status

In this study, we used CT images of 14 participants who were recruited for a previous study (3 men, 11 women, mean age 49.9, SD 11.9 years)^38^. Previously, OA status of the knees was assessed using a modified Kellgren–Lawrence (KL) OA severity scoring system^39^. Seven of 14 knees showed evidence of osteophyte and sclerosis and were classified as OA (1 M, 6 F; 52.4 ± 8.7 years; 101 ± 16 kg; 1 with KL = 1–2; 3 with KL = 2; 2 with KL = 3; 1 with KL = 4) while the remaining seven were classified as normal (2 M, 5 F; 47.3 ± 14.8 years; 72 ± 13 kg; KL = 0)^38^. The Institutional Research Board of the New England Baptist Hospital approved the study. All study procedures were conducted in accordance with the guidelines approved by the Institutional Research Board and the Declaration of Helsinki. Informed consent was obtained from all study participants.

### QCT Acquisition

Knee selection for CT imaging and imaging parameters were described previously^38^. If the participants had knee pain, the more painful knee was selected for imaging; otherwise, a random selection of left or right knee was scanned. Participants were scanned three times over two consecutive days. The knee of interest for each participant was imaged via single-energy QCT using a clinical CT scanner (Lightspeed 4-slice, General Electric, Milwaukee, WI, USA). Scanning was performed in the supine position of participants while the knee of interest was centered within the CT gantry. A solid QCT reference spine phantom (Model 3 T; Mindways Software Inc, Austin, TX, USA) was included in the images in order to convert grayscale CT Hounsfield units (HU) to equivalent apparent volumetric BMD (mg/cm^3^ K_2_HPO_4_). Scanned image volumes contained the distal femur, patella, proximal tibia, and fibula; though, image volumes were cropped to exclude the patella for this analysis. CT scanning parameters included: 120 kVp tube voltage, 150 mAs current-time product, axial scanning plane, 0.625 mm isotropic voxel size (0.625 mm slice thickness, 0.625 × 0.625 mm in-plane pixel size), ~240 slices, ~90 s scan time. Edge enhancement and post-processing were done using a standard bone reconstruction kernel (BONE). Effective radiation dose was estimated at ~0.073 mSv per scan using shareware software (CT-DOSE, National Board of Health, Herley, Denmark). This value is comparable to the average effective radiation dose during a transatlantic flight from Europe to North America (~0.05 mSv).

### CT Image Analysis

**Figure 3:**
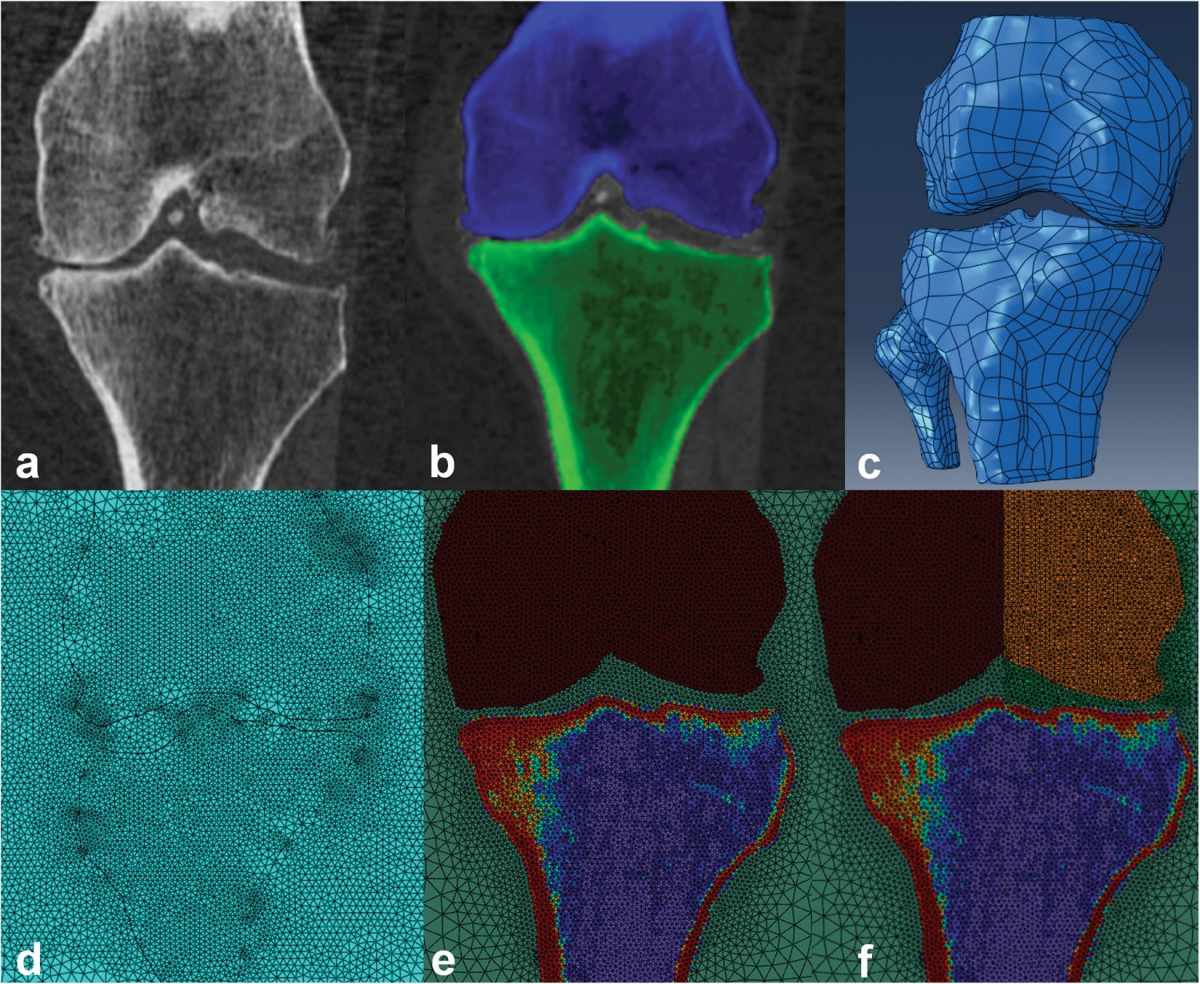
Methodological sequence for developing subject-specific FE model. (**a**) CT image of the knee. (**b**) Segmented bones of the knee. Image shows femur (blue) and tibia (green) in a coronal view. (**c**) Generated three-dimensional geometries of the femur, tibia, and fibula from CT images. (**d**) Meshed bones of the knee with 10-noded tetrahedral elements. Image shows femur, tibia, and soft tissue cylinder in the coronal plane. (**e**) Assigned material properties for the FE model. BMD was mapped to the modulus of elasticity of the bones. (**f**) To calculate the stiffness of medial compartment of the proximal tibia, the lateral compartment was isolated by assigning soft tissue material properties to the lateral distal femur.

We separated the proximal tibia, distal femur, and fibula from the surrounding soft tissue in the QCT images using semi-automatic segmentation and manual corrections. A subject-specific bone threshold, obtained using the half maximum height (HMH) technique, was used for segmenting each image^40,41^. In the HMH method, the density of a voxel with 50% cortical bone and 50% joint space is used as the minimum threshold for segmenting subchondral bone. We performed the segmentation using a commercial image processing software (ANALYZE10, Mayo Foundation, Rochester, MN, USA) (Fig. 3a,b), a stylus, and an interactive touch-screen tablet (Cintiq 21uX, Wacom, Krefeld, Germany).

### FE Modeling

#### Geometry

As CT imaging was performed in the supine position, the knees were reoriented in a neutral standing alignment (MATLAB, MathWorks, Natick, MA, USA) to model single-leg stance.

**Figure 4:**
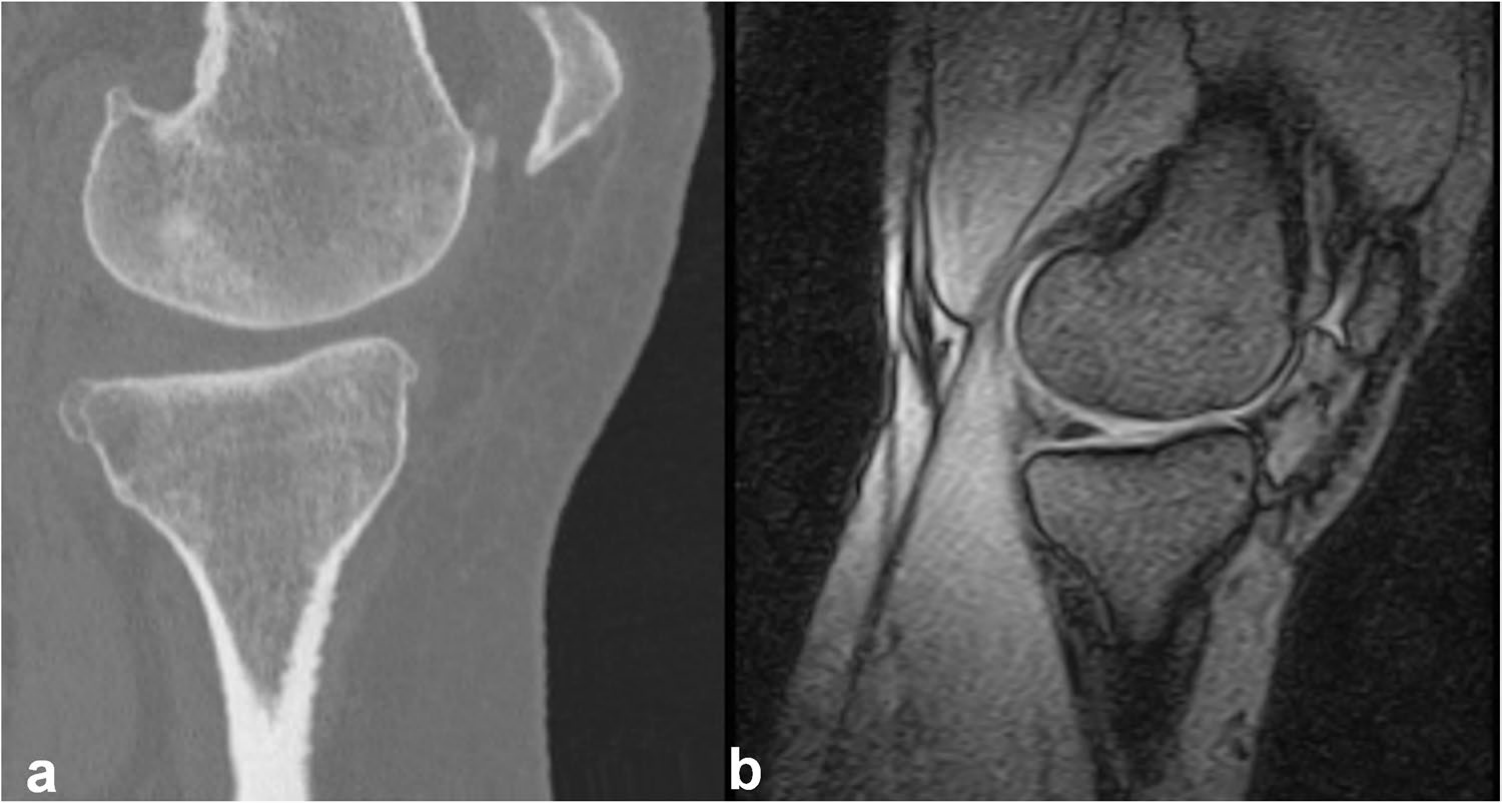
Comparison of re-aligned CT and generic standing MR position. (**a**) Re-aligned CT image and (**b**) Standing MR image. CT images were re-aligned such that the new alignments were similar to generic MR standing images.

Two vectors were defined for re-alignment; one vector was a best-fit line passing through the centroid of tibial cross-sections at different levels of the tibia (from distal to proximal: ankle; midshaft (50% length of tibia); 66% shaft site; proximal tibia). The other vector was a best-fit line passing through 32 femoral cross-sections starting just proximal to the intercondylar fossa and extending 2 cm proximally towards the center of the femoral head. The images were rotated such that the average of the two vectors was vertical. With this approach, both the femoral axis and the tibial axis make the same angle with the vertical axis (e.g., the varus angle was 175 °, the angle between the femoral axis and the vertical axis would be 2.5 °, and the same for the angle between the tibial axis and the vertical axis). Visually, the new alignment of the CT images was similar to generic standing MR images (Fig. 4).

We converted the segmented, re-aligned dataset to a 3D polygonal surface mesh using a marching cube algorithm (ANALYZE10, Mayo Foundation, Rochester, MN, USA) and imported the obtained 3D object into reverse engineering software (GEOMAGIC STUDIO 12, Systems, Rock Hill, SC, USA). We smoothed the surface of bones to ensure the geometry was topologically valid and did not contain holes or rough edges (Fig. 3c). To maintain geometric complexity, the maximum deviation of the smoothed surface from the original meshed surface was less than one voxel size (0.625 mm). We imported the resultant smoothed 3D volume of the knee into FE software (ABAQUS, Providence, RI, USA). Because soft tissues (e.g., cartilage, menisci) were indistinguishable in CT images, these tissues were modeled by an incompressible cylindrical medium, a method previously employed by McErlain *et al*.^22^. We then meshed all structures in the FE model using 10-noded, quadratic, tetrahedral elements (generic element size of 2 mm for bone and a generic element size of 20 mm for the soft tissue cylinder) (Fig. 3d). To identify an appropriate element size, we performed a mesh convergence study on seven different knee models. With this, FE results of stress and strain did not change by more than 1% when changing the element size from 2 mm to 1.8 mm. In total, FE models consisted of ~500000 to ~700000 elements depending on the size of the knees. Bonded contact was used between bone (the femur, tibia and fibula) and the soft tissue cylinder interface.

#### Material Properties

We converted imaged BMD to elastic modulus using a density-modulus equation proposed by Goulet *et al*.^32^ (Supplement Table 7). This equation has been shown to explain 70% of variance in proximal tibial subchondral bone stiffness^25^, and two studies recommended its use for modeling the proximal tibia^25,36^. We used a custom algorithm (MATLAB), reported by Nazemi *et al*.^25^, to map the material properties to tetrahedral elements^25^ (Fig. 3e). Elastic moduli ranged from 1 MPa to ~25 GPa for elements of the proximal tibia. Unfortunately, images of the distal femur were not full-length. This lead to localized, unrealistic load transfer from the most proximal cross-section of femur (where the load was applied) to the tibial subchondral bone (where the FE-results were analyzed) when using a flexible distal femur. To address this limitation, we modeled the distal femur as a rigid body with E set to 500 GPa. Since this is a common method used with subject-specific FE modeling^17,42,43^, and our analysis is focused on the proximal tibia, we believe this decision is justified. All of the elements of bone tissue were modeled with isotropic linear material properties and a Poisson’s ratio of 0.3^22^. For surrounding soft tissues, homogeneous, incompressible, and isotropic material properties were applied (E = 10 MPa, Poisson’s ratio = 0.495)^22^.

#### Loading and Boundary Conditions

We fixed the proximal femur in all directions except the longitudinal axis of the femur, where we applied a uniform displacement of 1 mm. The most distal sections of the tibia and fibula were constrained for all degrees of freedom. To normalize the results based on the weight of each participant, the vertical reaction force at the top surface of femur was obtained for each FE model in the FE software. As linear elastic models were used, the stress and strain results were adjusted based on the ratio of the derived reaction force to the weight of each participant. This is similar to applying one body weight to the most distal section of the proximal femur to simulate single-leg stance position.

### FE Outcomes

FE-based stiffness as well as minimum principal (the most compressive) and von-Mises stress and strain distributions were acquired for the proximal tibia. Although some argue that von-Mises stress and strain should not be used for analyzing bone^44^, we included these measures for comparison with previous research^22,26^. Different regions including peripheral cortical, subchondral cortical, subchondral trabecular, epiphyseal cortical, epiphyseal trabecular, metaphyseal cortical, and metaphyseal trabecular were defined for regional analysis of FE-based mechanical metrics (MATLAB) (Fig. 1). The regions were defined using a custom MATLAB code. The depth of each region was defined as per previous research^20,45^.

1. cortical, 0–2.5 mm from the outer surface of the tibia,
2. subchondral cortical, 0–2.5 mm from the surface of tibia in the plateau and spine,
3. subchondral trabecular, 2.5–5 mm from the surface of tibia in the plateau and spine,
4. peripheral, 0–5 mm depth from the tibial surface along the outer cortical region,
5. epiphyseal, 5–15 mm from the surface of tibia in the plateau and spine, and
6. metaphyseal, 15–35 mm from the surface of tibia in the plateau and spine.

Stiffness was calculated for each medial and lateral compartment by developing two additional models for each knee. The distal femur was divided into two compartments in these models, and a low elastic modulus (E = 10 MPa) was assigned to the opposite compartment of interest (i.e., when calculating medial proximal tibial stiffness, the lateral distal femur compartment was assigned a low elastic modulus) (Fig. 3f). This was done to ensure that all load was transferred to the compartment of interest in the proximal tibia (i.e., it avoided load sharing between the two compartments). Although this approach means that both halves of the tibia contribute (to some extent) to stiffness, we believe this stiffness measure is a reflective representation for individuals suffering from varus or valgus alignment, where all load could be transferred through either the medial or lateral compartment, respectively. Stiffness was calculated as the applied vertical load divided by the average vertical displacement of the subchondral bone surface nodes in the respective compartments.

### Statistical Analysis

We assessed short-term precision by calculating the CV%_RMS_ for each FE outcome as well as BMD, BMC and bone volume from 3 repeated scans of 14 individuals^34^. We compared minimum principal stress, von-Mises stress, minimum principal strain, von-Mises strain, and stiffness values in different regions of the proximal tibia between OA and normal knees using unpaired t-tests for normally distributed data and non-parametric Mann-Whitney U-tests for data that were not normally distributed. A variable with skewness or kurtosis Z-score outside of ±1.96 limits was considered to have a non-parametric distribution. We reported p-values and confidence intervals (CI) for each statistical test and considered an alpha level <5% to be statistically significant (IBM SPSS Statistics, Version 24.0. Armonk, NY, USA). 95% CI were reported for normal distributions while Hodges-Lehmann estimator was used to calculate confidence intervals for non-parametric distributions^46^. We also calculated Cohen’s *d* effect sizes for between-group mean differences in FE outcomes in relation to SD^47^. Cohen’s *d* > 0.8 was considered to be a large effect size with clinical significance^48^.

### Data Availability

The datasets used and analyzed during the current study are available from the corresponding author on reasonable request.

## Electronic supplementary material

Supplementary Information
