# Author Correction: Mechanical Metrics of the Proximal Tibia are Precise and Differentiate Osteoarthritic and Normal Knees: A Finite Element Study

**DOI:** 10.1038/s41598-019-43379-0

**Published:** 2019-05-02

**Authors:** Hanieh Arjmand, Majid Nazemi, Saija A. Kontulainen, Christine E. McLennan, David J. Hunter, David R. Wilson, James D. Johnston

**Affiliations:** 1 Department of Mechanical Engineering, University of Saskatchewan, Saskatoon, SK Canada; 2 College of Kinesiology, University of Saskatchewan, Saskatoon, SK Canada; 3 Division of Research, New England Baptist Hospital, Boston, MA USA; 4 Institute of Bone and Joint Research, Kolling Institute, University of Sydney and Rheumatology Department, Royal North Shore Hospital, Sydney, NSW Australia; 5 Department of Orthopaedics and Centre for Hip Health and Mobility, University of British Columbia and Vancouver Coastal Health Research Institute, Vancouver, BC Canada

Correction to: *Scientific Reports* 10.1038/s41598-018-29880-y, published online 31 July 2018

This Article contains errors in Tables 1 and 2 pertaining to sign convention. Specifically, minimum principal (compressive) strain measures in Table 2 were reported as positive whereas they should have been marked as negative, consistent with Table 1.

In addition, in Tables 1 and 2, under the column heading ‘Difference’,

“Absolute”

should read:

“Mean/Median*”

As a result, in the legends of Tables 1 and 2,

“(absolute and percent relative to normal)”

should read:

“(mean and percent relative to normal)”

The correct Tables 1 and 2 appear below with their accompanying legends.

**Table 1:** Minimum principal stress comparison between OA and normal proximal tibia.

Minimum principal stress (MPa)	All scans		CV%_RMS_	OA knees		Normal knees		Difference		95% CI		p-value	Cohen’s *d*
	Mean	SD		Mean/Median*	SD	Mean/Median*	SD	Mean/Median*	Percent	Lower	Upper		
Medial peripheral cortical *	−**0.36**	**0.19**	**5.7**	−**0.51**	**0.18**	−**0.22**	**0.08**	−**0.25**	**115.3%**	−**0.36**	−**0.11**	**0.006**	**1.36**
**Medial epiphyseal cortical**	−**0.59**	**0.31**	**6.6**	−**0.80**	**0.28**	−**0.38**	**0.17**	−**0.41**	**107.2%**	−**0.68**	−**0.14**	**0.006**	**1.33**
Medial metaphyseal cortical *	−**2.06**	**0.88**	**7.7**	−**2.56**	**0.95**	−**1.38**	**0.27**	−**1.18**	**85.2%**	−**1.71**	−**0.22**	**0.013**	**1.28**
Medial subchondral cortical *	−**0.52**	**0.16**	**3.9**	−**0.63**	**0.18**	−**0.41**	**0.06**	−**0.17**	**41.7%**	−**0.35**	−**0.02**	**0.018**	**1.22**
Medial subchondral trabecular *	−**0.46**	**0.15**	**3.7**	−**0.51**	**0.17**	−**0.37**	**0.06**	−**0.15**	**41.1%**	−**0.33**	−**0.01**	**0.035**	**1.13**
Medial epiphyseal trabecular *	−0.43	0.15	4.8	−0.47	0.18	−0.35	0.06	−0.12	33.2%	−0.32	0.01	0.085	1.03
Medial metaphyseal trabecular *	−0.51	0.26	8.9	−0.55	0.30	−0.34	0.10	−0.21	63.0%	−0.41	0.01	0.064	1.01
**Subchondral spine**	−**0.30**	**0.10**	**5.3**	−**0.37**	**0.07**	−**0.24**	**0.08**	−**0.14**	**56.9%**	−**0.22**	−**0.05**	**0.005**	**1.35**
Epiphyseal central	−0.14	0.06	8.2	−0.16	0.03	−0.14	0.07	−0.02	12.3%	−0.08	0.05	0.559	0.33
Metaphyseal central	−0.12	0.04	7.3	−0.13	0.03	−0.12	0.05	−0.01	3.8%	−0.05	0.04	0.838	0.12
Lateral subchondral cortical	−0.32	0.09	5.0	−0.35	0.11	−0.29	0.06	−0.07	22.8%	−0.17	0.03	0.175	0.74
Lateral subchondral trabecular	−0.25	0.07	4.6	−0.26	0.08	−0.25	0.06	−0.02	6.3%	−0.10	0.07	0.698	0.22
Lateral epiphyseal trabecular	−0.19	0.05	5.3	−0.20	0.06	−0.19	0.05	−0.01	3.0%	−0.07	0.06	0.847	0.11
Lateral metaphyseal trabecular	−0.15	0.04	10.5	−0.15	0.04	−0.16	0.06	0.00	2.2%	−0.05	0.06	0.896	0.07
Lateral peripheral cortical	−0.22	0.05	5.2	−0.24	0.05	−0.20	0.04	−0.04	22.4%	−0.10	0.01	0.104	0.87
Lateral epiphyseal cortical	−0.25	0.06	5.9	−0.29	0.06	−0.23	0.04	−0.06	25.2%	−0.12	0.00	0.057	1.00
Lateral metaphyseal cortical	−0.70	0.21	5.2	−0.76	0.25	−0.65	0.17	−0.11	17.1%	−0.36	0.14	0.346	0.53

Mean and SD of repeated scans for both OA and normal, CV%_RMS_, mean/median and SD for OA knees, mean/median and SD for normal knees, the difference between OA and normal knees (mean and percent relative to normal), 95% confidence of interval, p-value, and effect size (Cohen’s d) of minimum principal stress in different regions of proximal tibia. Measures with significant differences are shown with bold text in the table (p-value < 0.05).

(*) shows regions which were not normally distributed whereby median value used in Mann-Whitney U-tests for statistical comparison, and confidence intervals were calculated using Hodges-Lehmann estimator.

**Table 2:** Minimum principal strain comparison between OA and normal proximal tibia.

Minimum principal strain (microstrain)	All scans		CV%_RMS_	OA knees		Normal knees		Difference		95% CI		p-value	Cohen’s *d*
	Mean	SD		Mean/Median*	SD	Mean/Median*	SD	Mean/Median*	Percent	Lower	Upper		
Medial peripheral cortical	−1185	512	6.1	−1135	561	−1236	497	101	−8.2%	−516	+719	0.727	0.20
Medial epiphyseal cortical	−1410	601	4.2	−1435	666	−1385	582	−50	3.6%	−778	+678	0.883	0.08
Medial metaphyseal cortical	−1130	418	4.7	−1247	433	−1014	399	−233	23.0%	−718	+252	0.316	0.56
Medial subchondral cortical *	−563	203	5.6	−536	258	−512	143	−16	3.1%	−258	+164	0.949	0.28
Medial subchondral trabecular *	−770	322	6.5	−743	405	−627	189	−118	18.8%	−636	+155	0.338	0.64
Medial epiphyseal trabecular *	−2405	900	3.7	−2294	1133	−1990	599	−281	14.1%	−1403	+557	0.406	0.49
Medial metaphyseal trabecular	−2166	933	4.7	−2361	1096	−1970	771	−391	19.8%	−1494	+712	0.455	0.42
Subchondral spine *	−830	403	7.2	−749	544	−725	186	−27	3.7%	−817	+282	0.848	0.48
Epiphyseal central	−2688	879	3.2	−2873	1015	−2503	752	−370	14.8%	−1410	+670	0.454	0.42
Metaphyseal central	−1897	789	4.9	−2053	921	−1741	668	−312	17.9%	−1248	+625	0.482	0.39
Lateral subchondral cortical	−862	330	6.4	−914	410	−809	249	−106	13.1%	−500	+289	0.571	0.32
Lateral subchondral trabecular	−1321	447	5.2	−1495	548	−1148	249	−347	30.2%	−843	+149	0.154	0.78
Lateral epiphyseal trabecular *	−2508	860	3.9	−2434	1006	−1979	611	−559	28.2%	−1735	+346	0.180	0.71
Lateral metaphyseal trabecular	−1940	829	7.5	−2080	948	−1800	738	−280	15.6%	−1269	+709	0.549	0.34
Lateral peripheral cortical *	−1157	492	5.7	−933	657	−964	236	−10	1.0%	−953	+194	0.848	0.50
Lateral epiphyseal cortical	−1105	403	5.9	−1181	475	−1030	337	−151	14.7%	−631	+329	0.506	0.37
Lateral metaphyseal cortical	−670	288	7.4	−656	274	−685	323	30	−4.3%	−319	+378	0.857	0.10

Mean and SD of repeated scans for both OA and normal, CV%_RMS_, mean/median and SD for OA knees, mean/median and SD for normal knees, the difference between OA and normal knees (mean and percent relative to normal), 95% confidence of interval, p-value, and effect size (Cohen’s d) of minimum principal strain in different regions of proximal tibia.

(*) shows regions which were not normally distributed whereby median value used in Mann-Whitney U-tests for statistical comparison, and confidence intervals were calculated using Hodges-Lehmann estimator.

