# Supplementary material for: Mechanical Metrics of the Proximal Tibia are Precise and Differentiate Osteoarthritic and Normal Knees: A Finite Element Study
Source: Sci Rep. 2018 Jul 31;8:11478. doi: 10.1038/s41598-018-29880-y (PMC6068127; doi:10.1038/s41598-018-29880-y)
Supplement: Supplementary file 1 — Supplementary Information [file 41598_2018_29880_MOESM1_ESM.docx]

**Title:**  Mechanical Metrics of the Proximal Tibia are Precise and Differentiate Osteoarthritic and Normal Knees: A Finite Element Study

**Authors:** Hanieh Arjmand^1^; Majid Nazemi^1^; Saija A Kontulainen^2^; Christine E McLennan^3^; David J Hunter^4^; David R Wilson^5^; James D Johnston^1^

^1^Department of Mechanical Engineering, University of Saskatchewan, Saskatoon, SK, Canada

^2^College of Kinesiology, University of Saskatchewan, Saskatoon, SK, Canada

^3^Division of Research, New England Baptist Hospital, Boston, MA, USA

^4^Institute of Bone and Joint Research, Kolling Institute, University of Sydney and Rheumatology Department, Royal North Shore Hospital, Sydney, NSW, Australia

^5^Department of Orthopaedics and Centre for Hip Health and Mobility, University of British Columbia and Vancouver Costal Health Research Institute, Vancouver, BC, Canada

**Corresponding Author (& Requests Author)**

James (J.D.) Johnston

Department of Mechanical Engineering

University of Saskatchewan

57 Campus Dr.

Saskatoon, SK S7N 1G9 Canada

1-306-966-1468

[james.johnston@usask.ca](mailto:dena.burnett@usask.ca)

*Supplement Table 1- Von-Mises stress comparison between OA and normal proximal tibia.*

| von-Mises stress (MPa) | All scans | | CV%_RMS_ | OA knees | | Normal knees | | Difference | | 95% CI | | p-value | Cohen's *d* |
| --- | --- | --- | --- | --- | --- | --- | --- | --- | --- | --- | --- | --- | --- |
|  | Mean | SD |  | Mean/  Median* | SD | Mean/  Median* | SD | Absolute | Percent | Lower  limit | Upper  limit |  |  |
| **Medial peripheral cortical** | **0.42** | **0.19** | **4.9** | **0.59** | **0.17** | **0.28** | **0.08** | **0.28** | **101.3%** | **0.13** | **0.43** | **0.002** | **1.46** |
| **Medial epiphyseal cortical** | **0.71** | **0.38** | **6.1** | **0.96** | **0.35** | **0.45** | **0.20** | **0.51** | **112.6%** | **0.18** | **0.84** | **0.006** | **1.34** |
| **Medial metaphyseal cortical *** | **2.23** | **0.85** | **6.7** | **2.74** | **0.87** | **1.78** | **0.28** | **1.16** | **65.2%** | **0.26** | **1.74** | **0.025** | **1.31** |
| **Medial subchondral cortical** | **0.76** | **0.19** | **4.7** | **0.86** | **0.19** | **0.66** | **0.13** | **0.20** | **30.0%** | **0.01** | **0.39** | **0.042** | **1.06** |
| **Medial subchondral trabecular** | **0.64** | **0.17** | **4.2** | **0.74** | **0.18** | **0.54** | **0.09** | **0.20** | **37.2%** | **0.04** | **0.36** | **0.021** | **1.17** |
| **Medial epiphyseal trabecular *** | **0.45** | **0.15** | **4.8** | **0.50** | **0.18** | **0.37** | **0.06** | **0.13** | **35.0%** | **0.01** | **0.33** | **0.048** | **1.08** |
| Medial metaphyseal trabecular * | 0.55 | 0.26 | 8.9 | 0.59 | 0.30 | 0.37 | 0.11 | 0.23 | 62.1% | -0.01 | 0.46 | 0.064 | 1.04 |
| **Subchondral spine** | **0.34** | **0.11** | **4.7** | **0.43** | **0.07** | **0.27** | **0.08** | **0.16** | **60.4%** | **0.07** | **0.25** | **0.002** | **1.44** |
| Epiphyseal central | 0.15 | 0.06 | 7.9 | 0.17 | 0.03 | 0.14 | 0.07 | 0.03 | 25.1% | -0.03 | 0.10 | 0.281 | 0.60 |
| Metaphyseal central | 0.13 | 0.04 | 6.7 | 0.14 | 0.03 | 0.12 | 0.06 | 0.02 | 13.0% | -0.04 | 0.07 | 0.530 | 0.35 |
| Lateral subchondral cortical | 0.47 | 0.14 | 4.5 | 0.53 | 0.17 | 0.42 | 0.08 | 0.11 | 26.9% | -0.04 | 0.27 | 0.139 | 0.80 |
| Lateral subchondral trabecular | 0.34 | 0.11 | 4.8 | 0.37 | 0.14 | 0.31 | 0.07 | 0.06 | 17.6% | -0.07 | 0.18 | 0.368 | 0.50 |
| Lateral epiphyseal trabecular | 0.21 | 0.05 | 5.2 | 0.22 | 0.06 | 0.20 | 0.05 | 0.02 | 8.0% | -0.05 | 0.08 | 0.601 | 0.30 |
| Lateral metaphyseal trabecular | 0.16 | 0.04 | 10.3 | 0.17 | 0.04 | 0.16 | 0.05 | 0.01 | 7.4% | -0.04 | 0.07 | 0.651 | 0.26 |
| **Lateral peripheral cortical** | **0.29** | **0.06** | **4.4** | **0.33** | **0.05** | **0.26** | **0.04** | **0.08** | **30.5%** | **0.02** | **0.13** | **0.009** | **1.29** |
| **Lateral epiphyseal cortical** | **0.34** | **0.09** | **5.7** | **0.40** | **0.09** | **0.28** | **0.05** | **0.11** | **37.5%** | **0.02** | **0.19** | **0.018** | **1.20** |
| Lateral metaphyseal cortical | 0.86 | 0.24 | 4.1 | 0.94 | 0.29 | 0.78 | 0.16 | 0.17 | 21.3% | -0.11 | 0.44 | 0.209 | 0.69 |

*Mean and SD of repeated scans for both OA and normal, CV%_RMS_, mean and SD for OA knees, mean and SD for normal knees, the difference between OA and normal knees (absolute and percent relative to normal), 95% confidence of interval, p-value, and effect size (Cohen’s d) of von-Mises stress in different regions of proximal tibia. Measures with significant differences are shown with bold text in the table (p-value < 0.05).*

*(*) shows regions which were not normally distributed whereby median value used in Mann-Whitney U-tests for statistical comparison, and confidence intervals were calculated using Hodges-Lehmann estimator.*

*Supplement Table 2- Von-Mises strain comparison between OA and normal proximal tibia.*

| von-Mises strain (microstrain) | All scans | | CV%_RMS_ | OA knees | | Normal knees | | Difference | | 95% CI | | p-value | Cohen's *d* |
| --- | --- | --- | --- | --- | --- | --- | --- | --- | --- | --- | --- | --- | --- |
|  | Mean | SD |  | Mean  /Median* | SD | Mean  /Median* | SD | Absolute | Percent | Lower | Upper |  |  |
| Medial peripheral cortical | 1123 | 488 | 5.6 | 1077 | 531 | 1169 | 478 | -92 | -7.8% | -680 | 497 | 0.740 | 0.19 |
| Medial epiphyseal cortical | 1330 | 563 | 3.9 | 1368 | 635 | 1291 | 530 | 78 | 6.0% | -603 | 759 | 0.808 | 0.14 |
| Medial metaphyseal cortical | 1062 | 400 | 4.6 | 1167 | 427 | 956 | 373 | 211 | 22.0% | -256 | 677 | 0.344 | 0.53 |
| Medial subchondral cortical * | 550 | 203 | 4.9 | 508 | 260 | 515 | 142 | -7 | -1.4% | -162 | 257 | 0.949 | 0.23 |
| Medial subchondral trabecular * | 758 | 308 | 5.8 | 717 | 388 | 604 | 182 | 113 | 18.7% | -106 | 594 | 0.406 | 0.63 |
| Medial epiphyseal trabecular * | 2131 | 792 | 3.6 | 2018 | 995 | 1748 | 523 | 270 | 15.4% | -424 | 1224 | 0.482 | 0.51 |
| Medial metaphyseal trabecular | 1939 | 820 | 4.4 | 2113 | 962 | 1765 | 677 | 349 | 19.8% | -620 | 1318 | 0.448 | 0.43 |
| Subchondral spine * | 788 | 368 | 6.7 | 703 | 492 | 664 | 184 | 39 | 5.9% | -251 | 737 | 0.749 | 0.47 |
| Epiphyseal central | 2400 | 787 | 3.1 | 2587 | 917 | 2212 | 646 | 376 | 17.0% | -548 | 1300 | 0.393 | 0.48 |
| Metaphyseal central | 1728 | 706 | 4.4 | 1880 | 819 | 1575 | 595 | 306 | 19.4% | -528 | 1139 | 0.440 | 0.43 |
| Lateral subchondral cortical | 837 | 328 | 6.0 | 895 | 413 | 778 | 233 | 117 | 15.0% | -273 | 507 | 0.526 | 0.36 |
| Lateral subchondral trabecular | 1242 | 421 | 4.8 | 1405 | 514 | 1079 | 239 | 326 | 30.3% | -140 | 793 | 0.153 | 0.78 |
| Lateral epiphyseal trabecular * | 2273 | 781 | 3.7 | 2265 | 913 | 1815 | 555 | 450 | 24.8% | -296 | 1626 | 0.225 | 0.71 |
| Lateral metaphyseal trabecular | 1758 | 734 | 7.2 | 1888 | 832 | 1627 | 661 | 261 | 16.0% | -614 | 1136 | 0.528 | 0.36 |
| Lateral peripheral cortical * | 1130 | 481 | 5.1 | 929 | 638 | 951 | 228 | 33.4 | 3.5% | -179 | 943 | 0.565 | 0.55 |
| Lateral epiphyseal cortical | 1093 | 391 | 5.6 | 1176 | 466 | 1010 | 314 | 165 | 16.3% | -298 | 628 | 0.452 | 0.42 |
| Lateral metaphyseal cortical | 670 | 281 | 7.6 | 663 | 273 | 677 | 310 | -14 | -2.0% | -354 | 327 | 0.932 | 0.05 |

*Mean and SD of repeated scans for both OA and normal, CV%_RMS_, mean and SD for OA knees, mean and SD for normal knees, the difference between OA and normal knees (absolute and percent relative to normal), 95% confidence of interval, p-value, and effect size (Cohen’s d) of von-Mises strain in different regions of proximal tibia.*

*(*) shows regions which were not normally distributed whereby median value used in Mann-Whitney U-tests for statistical comparison, and confidence intervals were calculated using Hodges-Lehmann estimator.*

*Supplement Table 4- Bone mineral density (BMD) comparison between OA and normal proximal tibia.*

| Bone Mineral Density (g/cm^3^) | All scans | | CV%_RMS_ | OA knees | | Normal knees | | Difference | | 95% CI | | p-value | Cohen’s *d* |
| --- | --- | --- | --- | --- | --- | --- | --- | --- | --- | --- | --- | --- | --- |
|  | Mean | SD |  | Mean/Median* | SD | Mean/Median* | SD | Absolute | Percent | Lower | Upper |  |  |
| **Medial peripheral cortical** | **0.26** | **0.09** | **3.1** | **0.32** | **0.10** | **0.21** | **0.05** | **0.10** | **48.6%** | **0.01** | **0.20** | **0.039** | **1.11** |
| Medial epiphyseal cortical | 0.29 | 0.12 | 2.1 | 0.34 | 0.14 | 0.24 | 0.07 | 0.11 | 46.3% | -0.02 | 0.24 | 0.093 | 0.92 |
| Medial metaphyseal cortical | 0.57 | 0.14 | 3.6 | 0.62 | 0.17 | 0.53 | 0.10 | 0.09 | 16.6% | -0.07 | 0.25 | 0.254 | 0.64 |
| Medial subchondral cortical | 0.50 | 0.09 | 2.1 | 0.53 | 0.12 | 0.48 | 0.05 | 0.05 | 9.6% | -0.06 | 0.16 | 0.360 | 0.52 |
| Medial subchondral trabecular | 0.35 | 0.07 | 2.9 | 0.35 | 0.08 | 0.34 | 0.06 | 0.01 | 2.7% | -0.08 | 0.09 | 0.811 | 0.14 |
| Medial epiphyseal trabecular | 0.17 | 0.05 | 3.5 | 0.18 | 0.06 | 0.16 | 0.03 | 0.01 | 8.9% | -0.04 | 0.07 | 0.585 | 0.31 |
| Medial metaphyseal trabecular* | 0.18 | 0.06 | 5.9 | 0.15 | 0.08 | 0.15 | 0.05 | 0.01 | 6.9% | -0.04 | 0.11 | 0.535 | 0.43 |
| Subchondral spine | 0.32 | 0.06 | 2.5 | 0.35 | 0.07 | 0.28 | 0.04 | 0.07 | 23.3% | 0.00 | 0.13 | 0.050 | 1.04 |
| Epiphyseal central | 0.09 | 0.03 | 3.9 | 0.09 | 0.02 | 0.08 | 0.04 | 0.01 | 7.9% | -0.03 | 0.05 | 0.717 | 0.21 |
| Metaphyseal central | 0.09 | 0.04 | 9.1 | 0.09 | 0.04 | 0.09 | 0.05 | 0.00 | 4.3% | -0.05 | 0.06 | 0.881 | 0.09 |
| Lateral subchondral cortical | 0.36 | 0.07 | 3.2 | 0.38 | 0.09 | 0.34 | 0.05 | 0.03 | 9.4% | -0.06 | 0.12 | 0.443 | 0.43 |
| Lateral subchondral trabecular | 0.23 | 0.05 | 3.5 | 0.22 | 0.06 | 0.23 | 0.04 | -0.01 | -5.2% | -0.07 | 0.05 | 0.410 | 0.24 |
| Lateral epiphyseal trabecular | 0.12 | 0.03 | 4.3 | 0.11 | 0.03 | 0.12 | 0.03 | -0.01 | -11.0% | -0.05 | 0.02 | 0.669 | 0.46 |
| Lateral metaphyseal trabecular | 0.12 | 0.04 | 2.7 | 0.13 | 0.04 | 0.12 | 0.04 | 0.00 | 2.5% | -0.05 | 0.05 | 0.893 | 0.08 |
| Lateral peripheral cortical | 0.23 | 0.04 | 2.6 | 0.24 | 0.05 | 0.23 | 0.04 | 0.01 | 6.2% | -0.04 | 0.07 | 0.573 | 0.32 |
| Lateral epiphyseal cortical | 0.26 | 0.05 | 5 | 0.28 | 0.06 | 0.24 | 0.04 | 0.04 | 18.0% | -0.02 | 0.10 | 0.151 | 0.78 |
| Lateral metaphyseal cortical | 0.52 | 0.09 | 3.7 | 0.54 | 0.08 | 0.49 | 0.10 | 0.05 | 10.0% | -0.06 | 0.16 | 0.343 | 0.53 |

*Mean and SD of repeated scans for both OA and normal, CV%_RMS_, mean and SD for OA knees, mean and SD for normal knees, the difference between OA and normal knees (absolute and percent relative to normal), 95% confidence of interval, p-value, and effect size (Cohen’s d) of BMD in different regions of proximal tibia. Measures with significant differences are shown with bold text in the table (p-value < 0.05).*

*(*) shows regions which were not normally distributed whereby median value used in Mann-Whitney U-tests for statistical comparison, and confidence intervals were calculated using Hodges-Lehmann estimator.*

*Supplement Table 5 - Bone mineral content (BMC) comparison between OA and normal proximal tibia.*

| Bone mineral content (g) | All scans | | CV%_RMS_ | OA knees | | Normal knees | | Difference | | 95% CI | | p-value | Cohen’s *d* |
| --- | --- | --- | --- | --- | --- | --- | --- | --- | --- | --- | --- | --- | --- |
|  | Mean | SD |  | Mean | SD | Mean | SD | Absolute | Percent | Lower | Upper |  |  |
| Medial peripheral cortical | 0.44 | 0.18 | 4.7 | 0.53 | 0.19 | 0.35 | 0.13 | 0.18 | 50.7% | -0.37 | 0.01 | 0.063 | 0.99 |
| Medial epiphyseal cortical | 0.54 | 0.24 | 9.1 | 0.66 | 0.28 | 0.43 | 0.14 | 0.23 | 54.3% | -0.49 | 0.03 | 0.082 | 0.95 |
| Medial metaphyseal cortical | 1.53 | 0.31 | 7.9 | 1.62 | 0.33 | 1.44 | 0.28 | 0.19 | 12.9% | -0.54 | 0.17 | 0.280 | 0.60 |
| Medial subchondral cortical | 1.06 | 0.24 | 3.9 | 1.13 | 0.27 | 1.00 | 0.20 | 0.13 | 12.9% | -0.41 | 0.15 | 0.332 | 0.54 |
| Medial subchondral trabecular | 0.71 | 0.18 | 3.3 | 0.73 | 0.21 | 0.69 | 0.15 | 0.04 | 6.3% | -0.26 | 0.17 | 0.669 | 0.24 |
| Medial epiphyseal trabecular | 1.79 | 0.60 | 6.6 | 1.88 | 0.64 | 1.70 | 0.58 | 0.18 | 10.5% | -0.89 | 0.54 | 0.596 | 0.30 |
| Medial metaphyseal trabecular | 1.66 | 0.57 | 5.3 | 1.76 | 0.62 | 1.55 | 0.55 | 0.21 | 13.9% | -0.90 | 0.47 | 0.506 | 0.37 |
| Subchondral spine | 1.01 | 0.29 | 4.2 | 1.10 | 0.30 | 0.92 | 0.28 | 0.18 | 19.8% | -0.52 | 0.16 | 0.263 | 0.62 |
| Epiphyseal central | 0.89 | 0.41 | 5.5 | 0.91 | 0.18 | 0.87 | 0.57 | 0.04 | 4.5% | -0.53 | 0.45 | 0.868 | 0.10 |
| Metaphyseal central | 1.04 | 0.47 | 12.5 | 1.04 | 0.38 | 1.03 | 0.57 | 0.01 | 0.5% | -0.57 | 0.56 | 0.984 | 0.01 |
| Lateral subchondral cortical | 0.78 | 0.19 | 3.1 | 0.80 | 0.17 | 0.77 | 0.22 | 0.04 | 4.7% | -0.27 | 0.19 | 0.739 | 0.19 |
| Lateral subchondral trabecular | 0.48 | 0.13 | 2.9 | 0.46 | 0.10 | 0.50 | 0.16 | -0.05 | -9.1% | -0.11 | 0.20 | 0.540 | 0.35 |
| Lateral epiphyseal trabecular | 1.37 | 0.38 | 5.4 | 1.27 | 0.23 | 1.46 | 0.50 | -0.19 | -13.0% | -0.26 | 0.64 | 0.382 | 0.50 |
| Lateral metaphyseal trabecular | 1.58 | 0.79 | 11.3 | 1.53 | 0.57 | 1.62 | 1.01 | -0.09 | -5.8% | -0.86 | 1.05 | 0.834 | 0.12 |
| Lateral peripheral cortical | 0.32 | 0.07 | 3.8 | 0.33 | 0.07 | 0.31 | 0.07 | 0.02 | 4.9% | -0.10 | -0.37 | 0.684 | 0.23 |
| Lateral epiphyseal cortical | 0.58 | 0.12 | 8.2 | 0.63 | 0.08 | 0.54 | 0.14 | 0.09 | 17.3% | -0.23 | 0.04 | 0.170 | 0.76 |
| Lateral metaphyseal cortical | 1.83 | 0.34 | 9.0 | 1.91 | 0.31 | 1.75 | 0.38 | 0.16 | 9.1% | -0.56 | 0.24 | 0.403 | 0.47 |

*Mean and SD of repeated scans for both OA and normal, CV%_RMS_, mean and SD for OA knees, mean and SD for normal knees, the difference between OA and normal knees (absolute and percent relative to normal), 95% confidence of interval, p-value, and effect size (Cohen’s d) of BMC in different regions of proximal tibia.*

*Supplement Table 6 - Bone volume comparison between OA and normal proximal tibia.*

| Bone Volume (mm^3^) | All scans | | CV%_RMS_ | OA knees | | Normal knees | | Difference | | 95% CI | | p-value | Cohen’s *d* |
| --- | --- | --- | --- | --- | --- | --- | --- | --- | --- | --- | --- | --- | --- |
|  | Mean | SD |  | Mean | SD | Mean | SD | Absolute | Percent | Lower | Upper |  |  |
| Medial peripheral cortical | 1655 | 295 | 3.1 | 1675 | 252 | 1635 | 352 | 39 | 2.4% | -396 | 317 | 0.814 | 0.13 |
| Medial epiphyseal cortical | 1866 | 231 | 8.3 | 1909 | 148 | 1823 | 300 | 86 | 4.7% | -362 | 189 | 0.512 | 0.37 |
| Medial metaphyseal cortical | 2715 | 409 | 6.4 | 2691 | 413 | 2740 | 437 | -49 | -1.8% | -446 | 545 | 0.832 | 0.12 |
| Medial subchondral cortical | 2121 | 354 | 5.0 | 2156 | 340 | 2086 | 392 | 70 | 3.4% | -497 | 357 | 0.728 | 0.20 |
| Medial subchondral trabecular | 2055 | 346 | 4.6 | 2085 | 345 | 2026 | 371 | 59 | 2.9% | -476 | 358 | 0.763 | 0.17 |
| Medial epiphyseal trabecular | 10636 | 2165 | 8.5 | 10766 | 1949 | 10506 | 2514 | 261 | 2.5% | -2880 | 2359 | 0.832 | 0.12 |
| Medial metaphyseal trabecular | 9703 | 2963 | 9.3 | 9660 | 3201 | 9746 | 2961 | -86 | -0.9% | -3505 | 3678 | 0.959 | 0.03 |
| Subchondral spine | 3211 | 672 | 2.6 | 3189 | 727 | 3234 | 669 | -45 | -1.4% | -768 | 859 | 0.905 | 0.07 |
| Epiphyseal central | 10092 | 2392 | 3.7 | 10334 | 2562 | 9849 | 2387 | 485 | 4.9% | -3368 | 2399 | 0.720 | 0.20 |
| Metaphyseal central | 12358 | 2666 | 4.0 | 12425 | 2996 | 12291 | 2532 | 134 | 1.1% | -3364 | 3096 | 0.930 | 0.05 |
| Lateral subchondral cortical | 2213 | 452 | 4.6 | 2196 | 457 | 2229 | 483 | -32 | -1.4% | -515 | 579 | 0.900 | 0.07 |
| Lateral subchondral trabecular | 2161 | 459 | 4.3 | 2153 | 459 | 2169 | 494 | -16 | -0.8% | -539 | 572 | 0.950 | 0.04 |
| Lateral epiphyseal trabecular | 12217 | 2970 | 7.3 | 12357 | 3504 | 12078 | 2606 | 280 | 2.3% | -3876 | 3317 | 0.868 | 0.09 |
| Lateral metaphyseal trabecular | 12893 | 4952 | 10.8 | 12717 | 5014 | 13069 | 5285 | -352 | -2.7% | -5647 | 6351 | 0.900 | 0.07 |
| Lateral peripheral cortical | 1386 | 217 | 3.4 | 1378 | 168 | 1395 | 272 | -17 | -1.2% | -246 | 280 | 0.889 | 0.08 |
| Lateral epiphyseal cortical | 2305 | 388 | 7.2 | 2337 | 421 | 2273 | 383 | 64 | 2.8% | -532 | 405 | 0.771 | 0.16 |
| Lateral metaphyseal cortical | 3607 | 683 | 6.7 | 3601 | 742 | 3612 | 678 | -11 | -0.3% | -817 | 838 | 0.978 | 0.02 |

*Mean and SD of repeated scans for both OA and normal, CV%_RMS_, mean and SD for OA knees, mean and SD for normal knees, the difference between OA and normal knees (absolute and percent relative to normal), 95% confidence of interval, p-value, and effect size (Cohen’s d) of bone volume in different regions of proximal tibia.*

*Supplement Table 6- BMD^2.1^ comparison between OA and normal proximal tibia.*

| Bone Mineral Density ^2.1^ (g/cm^3^)^2.1^ | All scans | | CV%_RMS_ | OA knees | | Normal knees | | Difference | | 95% CI | | p-value | Cohen’s *d* |
| --- | --- | --- | --- | --- | --- | --- | --- | --- | --- | --- | --- | --- | --- |
|  | Mean | SD |  | Mean/Median* | SD | Mean/Median* | SD | Absolute | Percent | Lower | Upper |  |  |
| **Medial peripheral cortical*** | **0.19** | **0.14** | **5.3** | **0.21** | **0.16** | **0.13** | **0.04** | **0.10** | **46.9%** | **0.02** | **0.34** | **0.017** | **1.13** |
| Medial epiphyseal cortical* | 0.25 | 0.20 | 3.6 | 0.27 | 0.24 | 0.14 | 0.06 | 0.11 | 41.6% | 0.00 | 0.50 | 0.073 | 0.99 |
| Medial metaphyseal cortical | 0.86 | 0.35 | 6.4 | 0.98 | 0.43 | 0.75 | 0.23 | 0.23 | 23.5% | -0.19 | 0.65 | 0.247 | 0.65 |
| Medial subchondral cortical* | 0.58 | 0.20 | 3.1 | 0.55 | 0.26 | 0.54 | 0.10 | 0.02 | 3.6% | -0.10 | 0.38 | 0.710 | 0.56 |
| Medial subchondral trabecular | 0.28 | 0.10 | 4.4 | 0.29 | 0.13 | 0.26 | 0.08 | 0.03 | 9.4% | -0.10 | 0.16 | 0.643 | 0.26 |
| Medial epiphyseal trabecular * | 0.08 | 0.04 | 4.5 | 0.07 | 0.06 | 0.07 | 0.02 | 0.02 | 28.2% | -0.03 | 0.10 | 0.710 | 0.55 |
| Medial metaphyseal trabecular * | 0.10 | 0.08 | 7.6 | 0.08 | 0.11 | 0.06 | 0.04 | 0.01 | 18.7% | -0.02 | 0.11 | 0.535 | 0.56 |
| **Subchondral spine** | **0.29** | **0.11** | **3.5** | **0.35** | **0.11** | **0.22** | **0.05** | **0.13** | **37.2%** | **0.03** | **0.24** | **0.020** | **1.22** |
| Epiphyseal central | 0.03 | 0.01 | 5.1 | 0.03 | 0.01 | 0.03 | 0.02 | 0.00 | 8.4% | -0.01 | 0.02 | 0.713 | 0.21 |
| Metaphyseal central* | 0.04 | 0.02 | 14.0 | 0.04 | 0.02 | 0.04 | 0.02 | 0.01 | 11.9% | -0.02 | 0.03 | 0.805 | 0.13 |
| Lateral subchondral cortical* | 0.33 | 0.13 | 4.9 | 0.29 | 0.16 | 0.27 | 0.09 | 0.04 | 13.7% | -0.07 | 0.21 | 0.383 | 0.54 |
| Lateral subchondral trabecular | 0.13 | 0.05 | 5.8 | 0.13 | 0.06 | 0.14 | 0.05 | -0.01 | -7.7% | -0.07 | 0.05 | 0.729 | 0.20 |
| Lateral epiphyseal trabecular | 0.04 | 0.01 | 4.4 | 0.03 | 0.01 | 0.04 | 0.01 | -0.01 | -18.8% | -0.02 | 0.01 | 0.377 | 0.49 |
| Lateral metaphyseal trabecular | 0.06 | 0.03 | 7.4 | 0.07 | 0.03 | 0.06 | 0.03 | 0.01 | 8.7% | -0.03 | 0.04 | 0.738 | 0.19 |
| Lateral peripheral cortical | 0.15 | 0.05 | 5.6 | 0.17 | 0.06 | 0.14 | 0.05 | 0.03 | 19.8% | -0.03 | 0.09 | 0.241 | 0.65 |
| **Lateral epiphyseal cortical** | **0.20** | **0.08** | **10.6** | **0.24** | **0.08** | **0.16** | **0.05** | **0.08** | **33.4%** | **0.00** | **0.16** | **0.049** | **1.05** |
| Lateral metaphyseal cortical | 0.69 | 0.21 | 5.8 | 0.74 | 0.18 | 0.64 | 0.25 | 0.09 | 12.7% | -0.16 | 0.35 | 0.436 | 0.44 |

*Mean and SD of repeated scans for both OA and normal, CV%_RMS_, mean and SD for OA knees, mean and SD for normal knees, the difference between OA and normal knees (absolute and percent relative to normal), 95% confidence of interval, p-value, and effect size (Cohen’s d) of BMD^2.1^ in different regions of proximal tibia. Measures with significant differences are shown with bold text in the table (p-value < 0.05).*

*(*) shows regions which were not normally distributed whereby median value used in Mann-Whitney U-tests for statistical comparison, and confidence intervals were calculated using Hodges-Lehmann estimator.*

*Supplement Table 7 - Bone density and density-modulus equations.*

| $\text{E=6310}\left(\text{BV/TV}\right)^{2.1}$ | From Goulet et al. |
| --- | --- |
| $\text{ρ}_{\text{ash}}\text{=0.55ρ}_{\text{app}}$ | From Helgason et al. |
| $\text{ρ}_{\text{real}}\text{=1.8 g/cm}^{3}$ | From Carter et al. |
| $\text{ρ}_{\text{app}}\text{=ρ}_{\text{real}}\text{ × BV/TV}$ | From Helgason et al. |
| $\text{ρ}_{\text{ash}}\text{=1.06 BMD + 0.0389}$ | From Keyak et al. |

*Goulet’s density-modulus equation and density conversion relationships used in the FE models.*
